# Distribution and Predictors of Pesticides in the Umbilical Cord Blood of Chinese Newborns

**DOI:** 10.3390/ijerph13010094

**Published:** 2015-12-30

**Authors:** Monica K. Silver, Jie Shao, Minjian Chen, Yankai Xia, Betsy Lozoff, John D. Meeker

**Affiliations:** 1 Department of Environmental Health Sciences, University of Michigan, Ann Arbor, Michigan, MI 48109, USA; mksilver@umich.edu; 2 Department of Child Health Care, Children’s Hospital Zhejiang University School of Medicine, Hangzhou 310003, China; shaojie@zju.edu.cn; 3 Institute of Toxicology, Nanjing Medical University, Nanjing 210029, China; mjc1984@163.com (M.C.); yankaixia@njmu.edu.cn (Y.X.); 4 Center for Human Growth and Development, University of Michigan, Ann Arbor, Michigan, MI 41809, USA; blozoff@umich.edu

**Keywords:** pesticides, prenatal, pregnancy, infant, neonate, exposure assessment, China, cord blood

## Abstract

Rates of pesticide use in Chinese agriculture are five times greater than the global average, leading to high exposure via the diet. Many are neurotoxic, making prenatal pesticide exposure a concern. Previous studies of prenatal exposure in China focused almost entirely on organochlorines. Here the study goals were to characterize the exposure of Chinese newborns to all classes of pesticides and identify predictors of those exposures. Eighty-four pesticides and 12 metabolites were measured in the umbilical cord plasma of 336 infants. Composite variables were created for totals detected overall and by class. Individual pesticides were analyzed as dichotomous or continuous, based on detection rates. Relationships between demographic characteristics and pesticides were evaluated using generalized linear regression. Seventy-five pesticides were detected. The mean number of detects per sample was 15.3. Increased pesticide detects were found in the cord blood of infants born in the summer (β = 2.2, *p* = 0.01), particularly in July (β = 4.0, *p* = 0.03). Similar trends were observed for individual insecticide classes. Thus, a summer birth was the strongest predictor of pesticide evidence in cord blood. Associations were more striking for overall pesticide exposure than for individual pesticides, highlighting the importance of considering exposure to mixtures of pesticides, rather than individual agents or classes.

## 1. Introduction

Globally, nearly five million tons of synthetic pesticides are applied agriculturally each year [1,2]. China, one of the world’s largest consumers of pesticides [1,2,3], applies over 300,000 tons to food crops annually, more than 2.5- to 5-fold higher than the global average per field unit [4]. Rates in Zhejiang province, where this study was conducted, are some of the highest in China, at nearly double the national rate [5]. Farmers are thought to overuse or improperly use pesticides in an attempt to improve crop yields, resulting in high residual levels at the time of harvest [3,5].

Due to prolific pesticide use in agriculture, the most common route of non-occupational exposure to pesticides is via consumption of contaminated food [5]. Additional related exposures may also occur via contaminated drinking water and spray drift, especially in rural, farming communities, or from the use of residential pesticides in the home or yard [5]. Organophosphate (OP) insecticides are the most heavily used agricultural pesticide in China, while pyrethroid (PYR) insecticides are the most commonly used pesticides in residential settings [3].

Many pesticides, and particularly insecticides, act by disrupting signaling mechanisms in the central nervous system (CNS), thereby inhibiting neurological function. Because of their neurotoxic mode of action, pesticides have been implicated as possible contributors to the rise in neurodevelopmental disorders among children [6]. Infant and fetal brains are rapidly developing, making them vulnerable to the long-lasting effects of pesticide exposure, such as disruption of brain architecture or circuitry [7]. Pesticides are able to cross the placenta [8], and fetuses tend to have lower levels of detoxifying enzymes [9]. Both characteristics are thought to increase fetal susceptibility.

Despite having the world’s largest population coupled with the potential for high exposure, relatively little has been published about the levels of prenatal pesticide exposure in China. Five studies reported pesticide levels in umbilical cord blood [10,11,12,13,14], while others examined maternal urinary metabolites during pregnancy [4,15,16]. Of the five cord blood studies, only one measured pesticides of varying classes (insecticides, herbicides, fungicides, and repellants) [11]; all others focused solely on the organochlorine (OC) class of insecticides. 

Our exposure assessment extends these studies by examining 96 pesticides and metabolites from a wide variety of classes, enabling us to begin to consider the real-world problem of multiple, concurrent pesticide exposures. The goals of this study were to characterize pesticide exposure among Chinese newborns and identify predictors of exposure. This work lays the foundation for future work examining prenatal pesticide exposure and infant neurodevelopment in our cohort.

## 2. Methods

### 2.1. Ethics Statement

Institutional review board approval was obtained from the ethics committees of the University of Michigan (HUM00010107) and Children’s Hospital Zhejiang University. Signed, informed consent was obtained from parents.

### 2.2. Study Population

Pesticide analysis was performed for 336 infants from rural Fuyang county near Hangzhou, China in Zhejiang province. Pregnant women with healthy, uncomplicated, single pregnancies were recruited between 2008 and 2011 from Fuyang Maternal and Children’s Hospital at 37–42 weeks gestation and consented to cord blood screening (*n* = 1187). Of these infants, a subset (*n* = 359) was then enrolled in a study of iron deficiency and infant neurodevelopment. The subset for neurodevelopmental assessment was selected based on cord blood iron status (low, marginal, normal) and parental consent for the developmental study. Of those, 237 had a sufficient volume of cord blood available for pesticide analysis. The remaining pesticide analysis samples (*n* = 99) were randomly selected from those with sufficient cord blood volume from the original cord blood screening cohort.

### 2.3. Pesticides

Following delivery, 20–30 mL of cord blood was collected and immediately transferred from Fuyang to Hangzhou on dry ice. Samples were then separated and stored at −80 °C at Children’s Hospital Zhejiang University. Funding was obtained for the pesticide study in 2012. Plasma samples were transferred on dry ice to the Institute of Toxicology at Nanjing Medical University for pesticide analysis. Pesticides were chosen based on usage data, concerns of neurotoxicity, method compatibility, and pilot data. We analyzed 96 compounds (84 pesticides and 12 metabolites): 24 organophosphate (OP) insecticides, six OP metabolites, 12 pyrethroid (PYR) insecticides, one PYR metabolite, three carbamate (CARB) insecticides, five organochlorine (OC) insecticides, three OC metabolites, five miscellaneous insecticides of undetermined classes, 14 fungicides, two fungicide metabolites, 18 herbicides, and three “other-use” chemicals/synergists. 

The pesticide analysis protocol was modified from previously published methods [17,18]. Eight hundred-microliter plasma samples were mixed with 800 µL saturated ammonium sulfate. After centrifugation, the supernatant was subjected to solid-phase extraction (SPE) for cleaning and pre-concentration. The sample was loaded onto a conditioned and equilibrated ProElut C18 SPE cartridge (200 mg/3 mL; 50/pk, Dikma, China). After cleaning, analytes were harvested by eluting with dichloromethane and *n*-hexane. The SPE eluate was concentrated and reconstituted into 10 µL toluene prior to analysis. The pesticides in serum were then separated with a Thermo Scientific TRACE GC Ultra gas chromatograph equipped with a column of TR-PESTICIDE II (30 m, 0.25 mm, 0.25 µm) and measured in timed-SRM mode with a triple quadrupole TSQ XLS mass spectrometer (QqQ, Thermo Fisher Scientific, Inc., Waltham, MA, USA). Limits of detection (LODs) were determined by analyzing fortified serum on a signal-to-noise (S/N) ratio of three. Quality control samples were generated using serum samples with 0.675 and 1.35 ng/mL pesticide standards. Quality control samples and blanks were analyzed in parallel with study samples in each batch.

**Figure 1:**
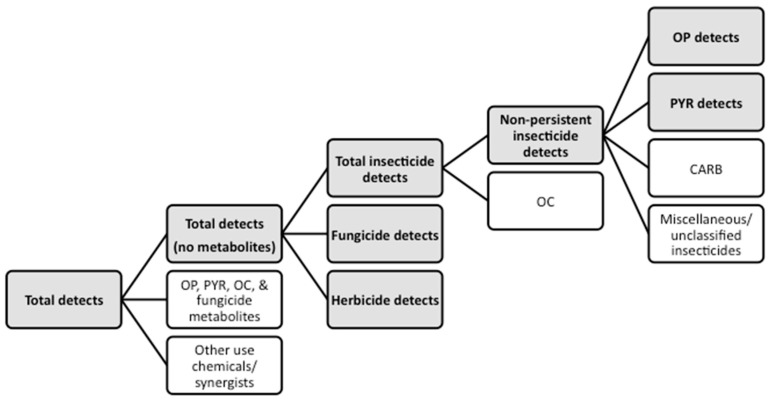
Creation of composite pesticide exposure variables ^1^. ^1^: Variables used in the analysis are shown in the gray boxes.

Given the likelihood of multiple concurrent pesticide exposures, we created several composite exposure variables. As a preliminary step, we dichotomized exposure to each pesticide. Concentrations below the limit of detection (<LOD) were assigned a value of 0, and those ≥LOD were assigned a value of 1. To assess overall pesticide exposure, we summed these dichotomous variables to create two indices of exposure for each infant: total number of pesticides detected and total number of pesticides detected not including metabolites. Because certain classes of pesticides may have similar modes of action and shared target sites within the body, we also created composites by class, summing the total number of exposures for each of the following: insecticides, non-persistent insecticides (no OCs), OPs, PYRs, fungicides, and herbicides (Figure 1).

Individual pesticides were also analyzed as continuous variables when detection rates were ≥80% (values <LOD were replaced with LOD/√2) or dichotomous variables (detect/non-detect), when detection rates were 10%–79%.

### 2.4. Predictors

Demographic and other variables analyzed as possible predictors of pesticide exposure were determined by maternal interview at the infant’s six-week follow-up visit. Household variables included: number of family members living in home, total number of people living in home, amount of living space in square meters, place of residence (rural/urban), and annual income (<30,000/30,000–49,999/50,000–99,999/≥100,000 Yuan). Parental characteristics included maternal and paternal age in years, education (middle school or less/high school or secondary school/college), and occupation (maternal: housewife/other; paternal: professional or administrator/manager/factory worker/other). Date of birth was used to create a season of birth variable (March–May/June–September/October–December) as well as a month of birth variable. All of the variables described here were analyzed as possible predictors of pesticide exposure.

### 2.5. Statistical Analysis

Statistical analyses were conducted using SAS 9.3 (SAS Institute Inc., Cary, NC, USA). Descriptive statistics and frequencies for all variables of interest were examined. Percentile tables were created to determine the individual pesticide distributions within the sample. Generalized linear models (GLM) were used to assess relationships between predictors and composite pesticide variables, as well as individual pesticides or metabolites with detection rates ≥80%. Logistic regression models were used to assess relations between predictors and individual pesticides or metabolites with detection rates 10%–79% (detected/non-detectable).

## 3. Results

**Figure 2:**
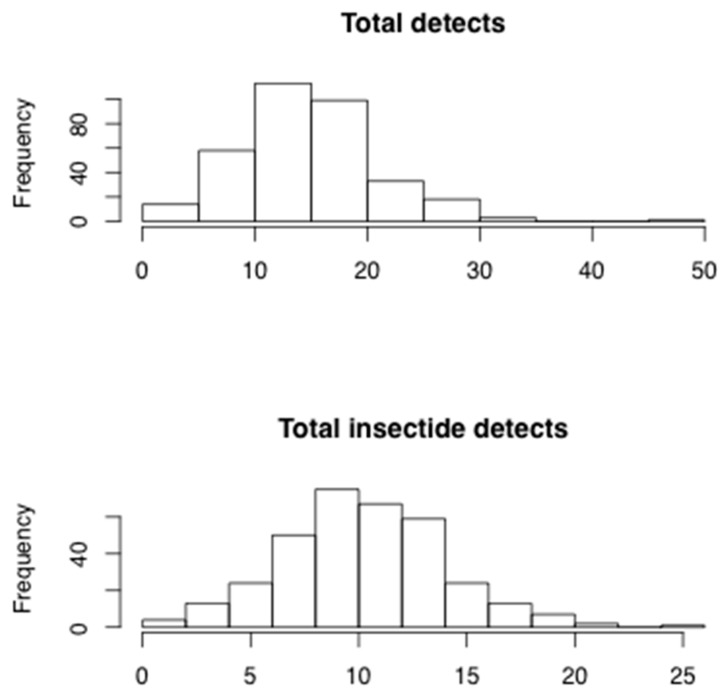
Distributions of number of detected pesticides and insecticides in cord blood plasma samples of infants from Zhejiang, China (*n* = 336) ^1^. ^1^: Histograms have different scales.

Seventy-five of the 96 pesticides and metabolites analyzed were detectable in at least one cord blood sample. The number of pesticides detected for individuals in the study population ranged from 0 to 48 with a mean (standard deviation (SD)) of 15.3 (6.1) (Figure 2). Excluding metabolites, the number of overall pesticides detected ranged from 0 to 41 with a mean (SD) of 12.5 (4.7). For total insecticides the range was 0 to 26 with a mean (SD) of 10.7 (3.7) (Figure 2). The distributions of all detectable pesticides and their LODs are shown in Table 1. LODs ranged but the majority were well below 1 ng/mL. Quality control analysis yielded coefficients of variation ranging from 5% to 34%.

**Table 1:** Distribution of pesticide concentrations in umbilical cord blood serum (ng/mL) at delivery, Zhejiang Province, China (*n* = 336).

			Selected Percentiles					
Pesticide	LOD	*n* > LOD (%)	50th	75th	90th	95th	99th	Max
*Organophosphates (OP)*								
Acephate	0.103	13 (3.9)	ND	ND	ND	ND	0.53	0.68
Chlorpyrifos	0.675	136 (40.5)	ND	0.96	3.85	6.24	9.08	11.40
Chlorpyrifos-methyl	0.005	20 (6.0)	ND	ND	ND	0.04	0.37	1.14
Diazinon	0.003	1 (0.3)	ND	ND	ND	ND	ND	0.38
Fensulfothion	0.033	1 (0.3)	ND	ND	ND	ND	ND	10.35
Fosthiazate	0.068	1 (0.3)	ND	ND	ND	ND	ND	7.82
Isofenphos-methyl	0.127	6 (1.8)	ND	ND	ND	ND	0.57	14.70
Methamidophos	1.519	218 (64.9)	4.23	24.54	63.85	115.94	231.96	496.86
Methidathion	0.068	1 (0.3)	ND	ND	ND	ND	ND	9.23
Mevinphos	0.123	27 (8.0)	ND	ND	ND	0.20	2.25	4.03
Monocrotophos	0.005	1 (0.3)	ND	ND	ND	ND	ND	0.05
Naled	0.422	312 (92.9)	1.77	5.14	11.06	20.03	50.31	74.68
Omethoate	1.350	116 (34.5)	ND	12.38	44.49	63.10	106.64	213.22
Phorate	1.789	103 (30.7)	ND	2.77	7.81	11.10	25.92	50.13
Terbufos	0.331	5 (1.5)	ND	ND	ND	ND	0.87	3.32
Trichlorfon	0.354	189 (56.3)	0.73	2.84	9.74	19.61	35.75	43.25
Carbophenothion sulfone ^m^	0.020	43 (12.8)	ND	ND	0.22	0.55	1.64	18.83
DEDTP ^m^	0.059	99 (29.5)	ND	0.43	1.09	1.55	2.44	3.63
DMDTP ^m^	1.738	21 (6.3)	ND	ND	ND	2.12	4.87	21.93
DMTP ^m^	1.350	1 (0.3)	ND	ND	ND	ND	ND	9.24
Phorate sulfone ^m^	0.006	6 (1.8)	ND	ND	ND	ND	0.04	1.19
TCPY ^m^	2.320	2 (0.6)	ND	ND	ND	ND	ND	13.52
*Pyrethroids (PYR)*								
Cyfluthrin	0.844	114 (33.9)	ND	1.49	3.06	4.00	10.55	1158.34
λ-Cyhalothrin	0.506	180 (53.6)	0.75	4.35	8.79	12.76	18.99	24.86
Cypermethrin	3.544	143 (42.6)	ND	7.58	14.53	20.39	35.55	390.27
Etofenprox	5.083	260 (77.4)	30.77	102.47	171.39	226.69	410.10	502.75
Fenpropathrin	0.053	150 (44.6)	ND	0.68	2.13	3.51	12.02	23.25
Fenvalerate	5.063	40 (11.9)	ND	ND	5.59	7.87	13.76	206.13
Flucythrinate	0.059	8 (2.4)	ND	ND	ND	ND	0.60	198.54
Fluvalinate-tau	6.075	46 (13.7)	ND	ND	9.15	15.87	27.02	39.60
*Cis*-Permethrin	2.194	252 (75.0)	6.92	15.57	28.32	39.28	278.91	470.05
*Trans*-Permethrin	0.030	239 (71.1)	2.81	124.10	314.95	449.97	596.69	737.84
Tefluthrin	0.174	7 (2.1)	ND	ND	ND	ND	0.21	0.95
Tetramethrin	3.881	59 (17.6)	ND	ND	12.45	20.22	33.74	41.48
3-Phenoxybenzoic acid ^m^	0.118	297 (88.4)	4.16	45.70	87.43	115.86	174.02	202.24
*Carbamates (CARB)*								
Pirimicarb	0.051	2 (0.6)	ND	ND	ND	ND	ND	0.41
Propoxur	0.008	334 (99.4)	0.13	12.73	29.00	35.29	49.44	58.66
*Organochlorines (OC)*								
Aldrin	0.135	276 (82.1)	1.66	3.90	7.04	11.36	28.16	40.75
Dicofol	2.536	28 (8.3)	ND	ND	ND	3.39	8.94	25.56
Dieldrin	5.569	31 (9.2)	ND	ND	ND	7.87	19.57	30.40
Mirex	0.003	77 (22.9)	ND	ND	0.07	0.16	1.59	120.00
Pentachlorophenol	0.007	22 (6.5)	ND	ND	ND	0.13	1.27	2.19
β-BHC ^m^	0.014	15 (4.5)	ND	ND	ND	ND	1.32	12.92
o,p’-DDE ^m^	0.036	88 (26.2)	ND	0.08	1.22	2.95	16.70	28.98
p,p’-DDE ^m^	0.034	121 (36.0)	ND	31.57	119.49	201.57	381.21	1101.73
*Miscellaneous/Unclassified Insecticides*								
Ethyl *p*-nitrophenyl thionobenzenephosphonate	0.482	7 (2.1)	ND	ND	ND	ND	3.56	16.14
Pyraclofos	0.042	1 (0.3)	ND	ND	ND	ND	ND	13.22
Prothiofos	5.063	149 (44.3)	ND	13.08	30.12	44.98	70.44	104.49
Pyridaben	0.304	26 (7.7)	ND	ND	ND	0.51	1.49	18.54
Spirodiclofen	0.037	3 (0.9)	ND	ND	ND	ND	ND	24.20
*Fungicides*								
Dicloran	0.003	1 (0.3)	ND	ND	ND	ND	ND	0.05
Difenoconazole	0.068	1 (0.3)	ND	ND	ND	ND	ND	13.40
Dimethomorph	0.014	1 (0.3)	ND	ND	ND	ND	ND	10.95
Furalaxyl	0.026	1 (0.3)	ND	ND	ND	ND	ND	11.13
Metalaxyl	0.014	68 (20.2)	ND	ND	0.22	0.42	0.61	0.66
Myclobutanil	0.068	1 (0.3)	ND	ND	ND	ND	ND	8.52
Nuarimol	0.014	1 (0.3)	ND	ND	ND	ND	ND	11.90
Oxadixyl	0.014	104 (31.0)	ND	0.32	1.25	4.74	17.83	60.07
Paclobutrazole	1.350	1 (0.3)	ND	ND	ND	ND	ND	10.56
Triadimefon	0.068	1 (0.3)	ND	ND	ND	ND	ND	9.76
Triflumizole	0.020	2 (0.6)	ND	ND	ND	ND	ND	8.86
Quinoxyfen	0.338	18 (5.4)	ND	ND	ND	0.35	4.21	35.43
Tetrahydrophthalimide ^m^	0.338	61 (18.2)	ND	ND	0.64	0.96	2.56	3.14
*Herbicides*								
Atrazine	0.008	25 (7.4)	ND	ND	ND	0.01	0.02	0.02
Barban	5.569	1 (0.3)	ND	ND	ND	ND	ND	8.25
Dicamba	1.266	6 (1.8)	ND	ND	ND	ND	1.87	2.74
Diphenamid	0.015	1 (0.3)	ND	ND	ND	ND	ND	9.09
2,4-Dichlorophenoxyacetic acid	0.506	73 (21.7)	ND	ND	1.26	1.80	4.05	58.24
Diuron	0.035	12 (3.6)	ND	ND	ND	ND	1.25	12.84
Fluridone	1.350	1 (0.3)	ND	ND	ND	ND	ND	10.85
Prometryn	0.020	208 (61.9)	1.20	7.50	17.34	29.30	64.12	182.18
Simazine	0.253	63 (18.8)	ND	ND	0.45	0.71	1.68	2.87
*Other-use chemicals/synergists*								
1-Hydroxynaphthalene	1.291	56 (16.7)	ND	ND	1.44	1.84	2.60	3.31
Piperonyl butoxide	0.219	14 (4.2)	ND	ND	ND	ND	1.30	16.57
Triphenylphosphate	0.068	111 (33.0)	ND	0.91	3.48	5.12	19.19	73.00

ND = non-detectable. ^m^ = denotes a metabolite. DEDTP = Diethyldithiophosphate; DMDTP = Dimethyldithiophosphate; DMTP = Dimethylthiophosphate; TCPY = 3,5,6-trichloro-2-pyridinol; β-BHC = β-Hexachlorohexane; o,p′-DDE = o,p′-*dichlorodiphenyldichloroethylene;* p,p′-DDE = p,p′-*dichlorodiphenyldichloroethylene.*

PYR and OP insecticides were the most commonly detected pesticides, with mean (SD) detects of 4.5 (2.1) and 3.4 (1.7), respectively. Complete distributions of pesticide concentrations are shown in Table 1. Propoxur, a CARB insecticide, was the most commonly detected pesticide, found in all but two of the cord blood samples. Three pesticides and one metabolite were detected in ≥80% of the samples (naled, propoxur, aldrin, 3-phenoxybenzoic acid). Undetected pesticides (by class) included: OP- dicrotophos, dimethoate, formothion, phosphamidon, dimethylvinphos, methyl parathion, malathion, dichlorvos; CARB- bendiocarb; fungicide- pyrimethanil, vinclozolin; fungicide metabolite- pentachloroaniline; herbicide- dimethipin, monolinuron, clomazone, isocarbamide, propyzamide, terbacil, dimethenamid, metribuzin, alachlor.

Demographic information was available for 237 infants. Characteristics of the study population are presented in Table 2. Two-thirds of the study population lived in a rural area. Around 40% of mothers and fathers had a middle school education or less. The most common maternal and paternal occupations were housewife and professional/administrative, respectively. Despite the majority living in a rural area, only 4% of families had at least one parent classified as a rural worker, which included farmers, forestry workers, fishermen, and animal caretakers. No infants were born during January or February since study enrollment did not occur around the Chinese New Year holiday.

**Table 2:** Family and household characteristics of the study population (*n* = 237).

Characteristics	*N*	Mean (SD)	Range
# Family in home	220	5.1 (1.3)	1–11
# People in home	210	4.3 (1.4)	1–9
Living space (square meters)	215	214.1 (147.9)	18–720
Maternal age (years)	216	26.1 (3.9)	18–41
Paternal age (years)	205	28.4 (4.4)	19–47
**Characteristics**	***N***	***N* (%)**	
Place of residence	216		
Rural		141 (65.3)	
City		75 (34.7)	
Annual income	215		
<30,000 Yuan		42 (19.5)	
30,000–49,999 Yuan		41 (19.1)	
50,000–99,999 Yuan		66 (30.7)	
≥100,000 Yuan		66 (30.7)	
Maternal education	221		
Middle school or less		84 (38.0)	
High school/secondary school		64 (29.0)	
College		73 (33.0)	
Paternal education	209		
Middle school or less		84 (40.2)	
High school/secondary school		57 (27.3)	
College		68 (32.5)	
Maternal occupation	221		
Housewife		91 (41.2)	
Other		130 (58.8)	
Paternal occupation	208		
Professional/Admin.		81 (38.9)	
Manager		32 (15.4)	
Factory worker		30 (14.4)	
Other		65 (31.3)	
Season of birth	237		
Spring (March–May)		62 (26.2)	
Summer (June–September)		91 (38.4)	
Fall/Winter (October–December)		84 (35.4)	

Infants born during the summer months of June to September had an average of 2.2 more pesticides detected in their cord blood than infants born between October and December (*p* = 0.01) (Table 3). By month of birth, July was the strongest predictor of overall pesticide exposure (4.0 more pesticides detected, on average, than for December, *p* = 0.03). No other household, demographic, or parental characteristics appeared to influence overall pesticide exposure in our population (Table S1).

**Table 3:** Selected results of generalized linear models for composite pesticide exposure variables, analyzing household, parental, and seasonal characteristics as predictors of exposure.

	Total Detects	Total Pesticides Detected (No Metabolites)	Total Insecticides Detected	Non-Persistent Insecticide Detected	OPs Detected	PYRs Detected	Fungicides Detected	Herbicides Detected
Predictor (Referent)	Effect Estimate (95% CI)	Effect Estimate (95% CI)	Effect Estimate (95% CI)	Effect Estimate (95% CI)	Effect Estimate (95% CI)	Effect Estimate (95% CI)	Effect Estimate (95% CI)	Effect Estimate (95% CI)
Paternal education (College)								
Middle school or less	−0.05 (−1.66–1.57)	−0.06 (−1.17–1.29)	0.07 (−0.92–1.06)	0.04 (−0.86–0.93)	0.03 (−0.43–0.48)	−0.03 (−0.58–0.52)	−0.01 (−0.27–0.24)	0.01 (−0.22–0.23)
High school/secondary school	−0.58 (−2.38–1.21)	−0.28 (−1.65–1.09)	−0.00 (−1.10–1.11)	−0.05 (−0.95–1.04)	−0.04 (−0.55–0.46)	0.01 (−0.60–0.62)	−0.29 (−0.57−0.01) *	0.01 (−0.25–0.26)
Maternal occupation (Housewife)								
Other	−0.84 (−2.18–0.49)	−0.73 (−1.75–0.29)	−0.66 (−1.48–0.16)	−0.66 (−1.40–0.08) ^†^	−0.33 (−0.71–0.05) ^†^	−0.28 (−0.74–0.19)	0.00 (−0.21–0.22)	−0.07 (−0.26–0.12)
Season of birth (Fall/Winter)								
Spring	0.64 (−1.02–2.30)	0.77 (−0.50–2.04)	0.65 (−0.38–1.67)	0.63 (−0.30–1.55)	0.00 (−0.48–0.47)	0.40 (−0.17–0.98)	−0.12 (−0.38–0.15)	0.24 (0.01–0.48) *
Summer	2.20 (0.52–3.88) *	1.59 (0.31–2.88) *	1.19 (0.15–2.22) *	1.13 (0.19–2.07) *	0.41 (−0.07–0.88) ^†^	0.63 (0.04–1.21) *	0.17 (−0.10–0.44)	0.24 (0.00–0.47) ^†^
Month of birth (December)								
March	−3.00 (−7.69–1.69)	−1.93 (−5.52–1.65)	−1.70 (−4.58–1.18)	−1.42 (−4.02–1.17)	−1.31 (−2.65–0.03) ^†^	0.06 (−1.55–1.66)	−0.46 (−1.21–0.29)	0.22 (−0.44–0.88)
April	0.44 (−2.70–3.58)	0.51 (−1.90–2.91)	−0.09 (−2.02–1.84)	−0.07 (−1.80–1.67)	−0.14 (−1.04–0.76)	0.01 (−1.06–1.09)	0.02 (−0.49–0.52)	0.58 (0.14–1.02) *
May	−0.45 (−3.73–2.83)	−0.27 (−2.78–2.23)	−0.37 (−2.37–1.64)	−0.48 (−2.28–1.34)	−0.45 (−1.39–0.49)	0.05 (−1.08–1.17)	−0.19 (−0.71–0.34)	0.28 (−0.18–0.74)
June	1.14 (−2.30–4.59)	0.70 (−1.94–3.33)	0.11 (−2.00–2.23)	0.25 (−1.66–2.16)	−0.10 (−1.08–0.89)	0.44 (−0.74–1.62)	0.07 (−0.48–0.62)	0.51 (0.02–0.99) *
July	4.04 (0.42–7.65) *	3.00 (0.24–5.76) *	2.15 (−0.07–4.37) ^†^	2.09 (0.09–4.09) *	0.57 (−0.46–1.61)	1.52 (0.28–2.76) *	0.11 (−0.47–0.69)	0.74 (0.23–1.25) **
August	1.31 (−2.34–4.95)	0.74 (−2.04–3.53)	0.12 (−2.12–2.35)	−0.15 (−2.16–1.87)	0.00 (−1.03–1.05)	0.06 (−1.19–1.30)	0.33 (−0.25–0.91)	0.30 (−0.21–0.81)
September	−0.38 (−3.85–3.08)	−0.63 (−3.28–2.02)	−1.12 (−3.25–1.01)	−1.16 (−3.08–0.75)	−0.14 (−1.13–0.85)	−0.80 (−1.98–0.39)	0.27 (−0.29–0.82)	0.22 (−0.27–0.71)
October	−1.47 (−4.90–1.96)	−1.08 (−3.71–1.54)	−1.39 (−3.50–0.72)	−1.25 (−3.15–0.65)	−0.69 (−1.68–0.29)	−0.25 (−1.42–0.92)	−0.14 (−0.69–0.41)	0.44 (−0.38–0.93) ^†^
November	−0.52 (−4.00–2.97)	−0.79 (−3.45–1.87)	−1.03 (−3.17–1.11)	−1.14 (−3.07–0.78)	−0.16 (−1.15–0.84)	−0.72 (−1.91–0.48)	0.20 (−0.36–0.76)	0.04 (−0.45–0.53)

** *p* < 0.01, * *p* < 0.05, ^†^ *p* < 0.10. Additional results can be found in Supplemental Table S1. CI= confidence interval.

Analyses of individual classes of pesticides similarly revealed that infants born in the summer had a higher number of pesticides detected in their cord blood (Table 3). Infants born between June and September had an average of 1.2 more insecticides, 0.4 more OPs, 0.7 more PYRs, and 0.2 more herbicides detected in their cord blood than infants born between October and December (*p* = 0.03, 0.09, 0.03, and 0.05, respectively). July was again the strongest predictor of insecticide exposure. Infants born in July had an average of 2.1 more insecticides, 1.5 more PYRs, and 0.7 more herbicides detected in their cord blood than infants born in December (*p* = 0.04, 0.02, and 0.004, respectively). There were also higher numbers of fungicides found in the cord blood of infants born in the spring, and the months of April, June, and October.

Significant predictors of highly detected (>50% detection) individual pesticides are shown in Table 4. Key findings are summarized here. On average, 3-phenoxybenzoic acid concentrations increased by 1.3 ng/mL for every year’s increase in maternal age (*p* = 0.05). Women who were not housewives had lower odds of detectable methamidophos, compared to housewives. Odds of detecting prothiophos were significantly higher in the spring, while the odds of detecting *trans*-permethrin, and trichlorfon were significantly higher in the summer, when compared to fall/winter. Naled concentrations were also significantly higher in the summer, while propoxur and 3-phenoxybenzoic acid concentrations were significantly lower in the spring, compared to the fall/winter. Additional significant findings for pesticides with lower detection rates (10%–50%) can be found in Table S2.

**Table 4:** Significant results of generalized linear models for individual pesticides with ≥80% detection rate and logistic regression models for pesticides with detection rates of 50%–80%, analyzing household, parental, and seasonal characteristics as predictors of exposure.

	Continuous Pesticide Results	Dichotomous Pesticide Results
Predictor (Referent)	Effect estimate (95% CI) ^1^	OR (95% CI) ^2^
Maternal age	3-Phenoxybenzoic Acid: 1.20 (0.05–2.34) *	
Maternal occupation (Housewife)		
Other		Methamidophos: 1.92 (1.20–3.08)
Season of birth (Fall/Winter)		
Spring	3-Phenoxybenzoic Acid: −21.54 (−33.50–9.60) ***	Prometryn: 0.44 (0.25–0.78)
	Propoxur: −7.19 (−10.62–3.76) ***	
	Aldrin: 1.37 (−0.16–2.89) ^†^	
Summer	Naled: 4.17 (1.42–6.92) **	Trichlorfon: 0.55 (0.31–0.95)
		*Trans*-permethrin: 0.49 (0.26–0.91)
Month of birth (December)		
March	Propoxur: −8.08 (−17.50–1.35) ^†^	
June	Aldrin: 3.07 (0.10–6.24) ^†^	
July	Naled: 8.81 (3.13–14.49) **	
September	3-Phenoxybenzoic Acid: 22.73 (−1.62–47.08) ^†^	
	Propoxur: 5.84 (−1.12–12.81) ^†^	
November	3-Phenoxybenzoic Acid: 25.95 (1.45–50.44) *	Prothiophos: 3.91 (1.15–13.28)
	Propoxur: 8.03 (0.99–15.07) *	

**^1^** = *** *p* < 0.001, ** *p* < 0.01, * *p* < 0.05, ^†^ *p* < 0.10; **^2^** = Modeled the probability that pesticide <LOD, so a value <1 means higher odds of detection, while a value >1 means lower odds of detection. CI= confidence interval. Additional results can be found in Supplemental Table S2.

## 4. Discussion

We found evidence of prenatal exposure to 75 pesticides or pesticide metabolites in a cohort of Chinese newborns in Zhejiang Province. Neonates, on average, had detectable levels of 15 pesticides in their cord blood. Season of birth, specifically summer, was the strongest predictor of increased number of pesticides detected in cord blood. Infants born in July had a significantly greater incidence of cord pesticides than infants born in December. Similar trends were observed for individual classes of insecticides.

Until now, no study of this scope has been completed in China or elsewhere. A few relevant studies in the U.S. have measured 14–29 pesticides in all classes in cord blood [19,20,21]. Previous Chinese studies using cord blood analyzed a limited number of pesticides within a single class, reporting levels of 6–18 OC insecticides [10,12,13,14]. Only our own pilot study reported cord blood levels for mixed classes (OPs, CARBs, herbicides, and fungicides) [11]. Chinese studies of other common classes of insecticides, such as OPs and PYRs, have used maternal non-specific urinary metabolites during pregnancy as biomarkers of prenatal exposure [4,15,16]. 

Of the pesticides measured in the present study, 29 were measured in previous U.S. [19,20,21] or Chinese studies [10,11,12,13,14]. Table 5 compares the high ends of the exposure distributions across the studies. In general, the 90th percentile concentrations in the Chinese studies are several orders of magnitude higher than the maximums reported in the U.S. studies. For example, for *cis*-permethrin, a common pyrethroid insecticide, the 90th percentile for the current study was 28.32 ng/mL, while the comparable U.S. values were 0.001–0.010 ng/mL [19,20,21]. The pattern was similar for all pyrethroids, as well as many other pesticides. Thus, it appears that some infants in our study population were prenatally exposed to very high levels or pesticides compared to U.S. infants. However, we did not detect exposure to certain pesticides reported in U.S. or other Chinese studies. These included dichlorvos, malathion, methyl parathion, bendiocarb, vinclozilin, and alachlor. Dichlorvos and bendiocarb have never been measured in China before and may not be used there, or perhaps our methods were not sufficiently sensitive to detect them. Malathion, methyl parathion, vinclozilin, and alachlor were previously detected in our pilot work [11]. It is unclear why they were not detected with updated analytical methods.

**Table 5:** Comparison of cord blood serum or plasma samples from the current study and previously published studies in the U.S. and China (ng/mL).

Pesticide	Current Study ^1^	U.S. Studies ^2^	Chinese Studies ^1^
***Organophosphates (OP)***			
Chlorpyrifos	3.85	0.014 [19]; 0.002 [20]; 0.065 [21] *	0.17 [11]
Diazinon	0.38 ^max^	ND [19]; 0.003 [20]; 0.013 [21] *	0.27 [11]
Dichlorvos	ND	0.005 [21] *	NM
Malathion	ND	0.048 [21] *	3.13 [11]
Methyl parathion	ND	0.016 [21] *	1.43 [11]
Phorate	7.81	0.010 [21] *	NM
Terbufos	3.32 ^max^	0.071 [21] *	0.27 [11]
***Pyrethroids (PYR)***			
Cyfluthrin	3.06	0.084 [19]	NM
Cyhalothrin	8.79	ND [19]	NM
Cypermethrin	14.53	ND [19]	NM
Fenvalerate	5.59	ND [19]	NM
*Cis*-Permethrin	28.32	0.010 [19]; 0.001 [20]; 0.004 [21] *	NM
*Trans*-Permethrin	314.95	0.028 [19]; 0.002 [20]; 0.005 [21] *	NM
Tetramethrin	12.45	ND [19]	NM
***Carbamates (CARB)***			
Bendiocarb	ND	0.032 [21] *	NM
Propoxur	29.00	0.033 [19]; 0.670 [21] *	0.19 [11]
***Organochlorines (OC)***			
Aldrin	7.04	NM	5.56 ^max,^ [13]
Dieldrin	7.87 ^95th^	NM	20.79 ^max,^ [13]
Mirex	0.07	0.031 [19]	0.16 ^max,^ [14] *
β-BHC ^m^	12.92 ^max^	NM	9.69 ^max,^ [13]; 0.09 ^med,^ [10] *; 1.79 ^max,^ [14] *; 0.07, 0.33, 0.14 ^max,^ [12] *
o,p′-DDE ^m^	1.22	NM	0.03 ^max,^ [14]; ND ^med,^ [10] *
p,p′-DDE ^m^	119.49	17.734 [19]	31.66 ^max,^ [13]; 1.32 ^med,^ [10] *; 17.16 ^max,^ [14] *; 1.37, 9.76, 85.19 ^max,^ [12] *
***Fungicides (FUNG)***			
Dicloran	0.05 ^max^	0.033 [21] *	4.73 [11]
Metalaxyl	0.22	0.015 [20]; 0.258 [21] *	18.60 ^max,^ [11]
Vinclozolin	ND	NM	0.94 [11]
Tetrahydrophthalimide ^m^	0.64	0.014 [20]; 0.038 [21] *	
***Herbicides (HERB)***			
Alachlor	ND	0.015 [21] *	2.21 [11]
Atrazine	0.01 ^95th^	0.012 [21] *	1.47 [11]
***Non-pesticides***			
Piperonyl butoxide	16.57 ^max^	0.0001 [19]	NM

ND = non-detectable. NM = not measured. ^m^ = denotes a metabolite. **^1^** = 90th percentile concentrations are shown unless otherwise indicated. **^2^** = Maximum concentrations are shown unless otherwise indicated. ^max^ = Maximum; ^95th^ = 95th percentile; ^90th^ = 90th percentile; ^med^ = Median. * Denotes an estimated value: [21]- data was estimated by converting from pg/g plasma to ng/mL by multiplying by 1.03/1000 (weight of plasma is 1.03 g/mL; there are 1000 pg per ng). [14], [10], and [12]- data was estimated by converting from ng/g lipid to ng/mL (non-lipid adjusted) by multiplying by 6.84/1000 (calculated average of lipid concentration as 6.84 g lipid/L**; there are 1000 ml per L). ** Reported levels of triglyceride (TG) = 3.02 mmol/L and cholesterol, LDL = 3.07 mmol/L and HDL = 1.76 mmol/L in Hangzhou infant cord blood [22]. We converted these values to mg/dl and determined total lipids using the equation: total lipid (g/L) = 0.9 + 1.3(chol (g/L) + TG (g/L)) [10,23]. Using this equation, we estimated the total lipid concentration in Chinese infants to be 6.84 g lipid/L.

Several prior studies also analyzed demographic characteristics as predictors of OC or PYR exposure in China. An exposure assessment of OCs in women of childbearing age reported lower OC levels in women with higher income and education [24], while a study of OCs in cord blood found the opposite [13]. Two studies of PYR exposure in pregnant women found that maternal education was inversely related to PYR, with PYR urinary metabolites decreasing with higher education level [15,16]. Positive associations between PYR metabolites and work as a manual laborer were reported for both studies [15,16] and with being a housewife in one study [16]. In contrast, we did not observe any significant associations between overall cord pesticide levels, OC, or PYR exposure and either income or maternal education. The number of fungicides detected was slightly lower in infants whose fathers had a secondary school *versus* college education, but this may be a chance finding. There were no noticeable trends of pesticide exposure by category of parental occupation, though non-housewives were slightly less exposed overall to OPs and had lower odds of detection for methamidophos. However, we had to rely on relatively broad, non-specific occupation categories and a dichotomous exposure metric (detect/non-detect). Finally, we did not find higher exposure in rural *versus* urban areas, in contrast to a previous study of PYR exposure [15]. No previous studies analyzed predictors of OP, CARB, herbicide, or fungicide prenatal exposure in China. 

Season of birth is a relatively unexplored predictor of prenatal pesticide exposure in China. One previous study reported higher levels of PYR urinary metabolites in pregnant women in June through September [16], and pesticide poisonings are most commonly reported in August and September in Zhejiang province, which coincides with the farming season [25]. In our study, season of birth was the strongest and most consistent predictor of cord pesticides. The total number of pesticides detected, the total insecticides, and the total OPs, PYRs, and fungicides detected were all higher in the cord blood of infants born in the summer months of June to September, compared to those born between October and December. Findings for individual pesticides also varied significantly by season and specific month. Although we were unable to find any data on seasonal or monthly pesticide usage in China, it seems likely that these levels correlate with typical time of pesticide applications both agriculturally and residentially. 

There were some additional limitations to this work. Because we measured a large number of pesticides with widely varying properties, the methods were not fully optimized for certain pesticides or classes of pesticides. This likely resulted in higher detection limits for some pesticides, compared to a more targeted approach. We were also unable to quantify exposure to some common pesticides and metabolites of interest, due to limitations in optimizing this high-throughput methodology to all chemicals of interest. Generally speaking, analysis of pesticides in blood can result in a high frequency of non-detection, since pesticide levels in blood tend to be low, particularly for non-persistent pesticides that are rapidly metabolized [26]. While our optimized GC-MS/MS methods helped to minimize this concern, we still had many cases of non-detection, limiting our ability to analyze pesticides on an individual basis, and necessitating the use of crude measures of exposure, such as number of detects by pesticide class. This approach is limited because it may not reflect the dose or level of exposure. Additionally, funding for pesticide analyses was not received until a year after cord blood collection was completed. Pesticides are biologically reactive and may break down over time [26,27], although our blood collection and storage protocols were carefully designed to minimize these effects. Similarly, most of the pesticides measured here, with the exception of OC insecticides, were non-persistent. With only one measure of exposure, we were unable to address the temporal variability of exposure during pregnancy. Another limitation is that we did not have data on lipid levels to adjust OC insecticide concentrations, as is the standard. Furthermore, because this was not originally designed as an environmental exposure study, we did not have information about residential pesticide use, maternal diet during pregnancy, and proximity to agriculture, which would have made this study more robust. Infants were not enrolled during the Chinese New Year holiday season (January–February), which limits our data on prenatal pesticide exposures during those winter months. Finally, the pesticide levels reported here for our infants from Zhejiang Province may not be representative of newborns elsewhere in China.

Despite its limitations, this study has important strengths. To our knowledge, it is the largest and most comprehensive exposure assessment of prenatal pesticide exposure anywhere in the world to date. The use of umbilical cord blood, as opposed to non-specific urinary metabolites, provides unequivocal evidence of fetal exposure [26,27] and may be more likely to reflect the available dose, since the measured dose was not yet eliminated from the infant’s body [28]. These considerations are relevant for assessing and managing risk. Our analysis of predictors of prenatal exposure is more comprehensive than in previous Chinese studies. Our findings that associations between season of birth and exposure were more striking for overall pesticide exposure than for individual pesticides provide an important first step in highlighting the importance of considering exposures to mixtures of chemicals, rather than focusing solely on individual agents or classes.

## 5. Conclusions

In conclusion, we reported pesticide exposure profiles in cord blood for 336 Chinese infants. Seventy-five of 96 possible pesticides/metabolites were detected in at least one sample. On average, the infants had 15 pesticides detected in their cord blood samples, with some having as many as 48. Infants born in the summer months, especially in July, had greater numbers of detected pesticides in their cord blood, compared to infants born in the winter. Levels for many of the pesticides measured here, and particularly the pyrethroid insecticides, were orders of magnitude higher than those reported in cord blood in U.S. studies. Prenatal pesticide exposure is a concern, because the fetal brain is rapidly developing *in utero* and pesticide exposure during this period of critical development may have long-lasting effects on neurodevelopment. Many of the pesticides included in this analysis are proven or suspected developmental toxicants and future work in this cohort will seek to further elucidate the relationships between prenatal pesticide exposure and infant neurodvelopment. Although Chinese infants may be some of the most highly exposed in the world, due to high rates of pesticide use in Chinese agriculture, the pesticides targeted in this study are used worldwide.

## Supplementary Files

Supplementary File 1
