# Supplementary material for: Distribution and Predictors of Pesticides in the Umbilical Cord Blood of Chinese Newborns
Source: Int J Environ Res Public Health. 2015 Dec 30;13(1):94. doi: 10.3390/ijerph13010094 (PMC4730485; doi:10.3390/ijerph13010094)
Supplement: Supplementary File 1 [file ijerph-13-00094-s001.pdf]

Supplementary Materials: Distribution and Predictors of Pesticides in the Umbilical Cord Blood of Chinese Newborns

Monica K. Silver, Jie Shao, Minjian Chen, Yankai Xia, Betsy Lozoff and John D. Meeker

Table S1. Additional results of generalized linear models for composite pesticide exposure variables, analyzing household, parental, and seasonal characteristics as predictors of exposure.

	Total Detects	Total Detects (No Metabolites)	Total Insecticide Detects	Non-Persistent Insecticide Detects	OP Detects	PYR Detects	Fungicide Detects	Herbicide Detects
**Predictor (Referent)**	**Effect Estimate (95% CI)**	**Effect Estimate (95% CI)**	**Effect Estimate (95% CI)**	**Effect Estimate (95% CI)**	**Effect Estimate (95% CI)**	**Effect Estimate (95% CI)**	**Effect Estimate (95% CI)**	**Effect Estimate (95% CI)**
# Family in home	−0.12 (−0.63–0.39)	−0.05 (−0.44–0.33)	−0.07 (−0.39–0.24)	−0.08 (−0.36–0.20)	0.03 (−0.12–0.17)	−0.12 (−0.30–0.05)	−0.02 (−0.10–0.06)	0.04 (−0.04–0.11)
# People in home	0.04 (−0.42–0.50)	0.05 (−0.31–0.40)	0.02 (−0.26–0.31)	0.00 (−0.26–0.26)	0.08 (−0.05–0.21)	−0.08 (−0.24–0.08)	0.02 (−0.05–0.10)	0.00 (−0.07–0.07)
Living space	0.00 (−0.01–0.00)	0.00 (−0.00–0.00)	0.00 (−0.00–0.00)	0.00 (−0.00–0.00)	0.00 (−0.00–0.00)	0.00 (−0.00–0.00)	0.00 (−0.00–0.00)	0.00 (−0.00–0.00)
Residence (Urban)								
Rural	−0.06 (−1.47–1.35)	0.04 (−1.03–1.12)	0.01 (−0.86–0.87)	0.03 (−0.75–0.81)	−0.09 (−0.49–0.31)	0.10 (−0.39–0.58)	−0.01 (−0.24–0.21)	0.08 (−0.12–0.27)
Income (≥100,000 Yuan)								
<30,000 Yuan	−0.49 (−2.42–1.44)	−0.46 (−1.93–1.01)	−0.21 (−1.39–0.97)	−0.15 (−1.22–0.91)	−0.01 (−0.55–0.54)	−0.15 (−0.81–0.52)	−0.04 (−0.34–0.27)	−0.21 (−0.49–0.06)
30,000–49,999 Yuan	0.44 (−1.59–2.47)	0.07 (−1.48–1.61)	0.23 (−1.01–1.47)	0.08 (−1.04–1.19)	0.26 (−0.31–0.84)	−0.27 (−0.97–0.43)	0.03 (−0.30–0.35)	−0.19 (−0.47–0.10)
50,000–99,999 Yuan	0.28 (−1.59–2.46)	0.15 (−1.26–1.56)	0.06 (−1.07–1.20)	0.02 (−1.00–1.04)	−0.08 (−0.61–0.44)	0.01 (−0.65–0.63)	0.17 (−0.13–0.47)	−0.08 (−0.34–0.18)
Maternal age	0.05 (−0.13–0.24)	0.02 (−0.13–0.16)	0.00 (−0.11–0.12)	−0.01 (−0.12–0.09)	0.02 (−0.04–0.07)	−0.02 (−0.09–0.04)	0.02 (−0.01–0.05)	−0.01 (−0.04–0.02)
Paternal age	−0.06 (−0.22–0.10)	−0.05 (−0.18–0.07)	−0.04 (−0.14–0.05)	−0.03 (−0.12–0.05)	0.00 (−0.05–0.04)	−0.02 (−0.07–0.03)	−0.00 (−0.03–0.02)	−0.01 (−0.03–0.02)
Maternal education (College)								
Middle school or less	0.14 (−1.42–1.70)	0.14 (−1.06–1.33)	0.26 (−0.70–1.22)	0.25 (−0.62–1.11)	0.04 (−0.40–0.48)	0.13 (−0.41–0.66)	−0.03 (−0.27–0.22)	−0.10 (−0.32–0.12)
High school/secondary school	0.12 (−1.58–1.82)	0.32 (−0.98–1.62)	0.37 (−0.67–1.41)	0.42 (−0.53–1.36)	0.20 (−0.29–0.68)	0.12 (−0.47–0.70)	−0.05 (−0.32–0.22)	0.00 (−0.24–0.24)
Paternal occupation (Prof./Tech./Admin.)								
Manager	0.21 (−1.77–2.19)	0.04 (−1.47–1.56)	−0.15 (−1.36–1.07)	−0.13 (−1.23–0.97)	−0.06 (−0.63–0.49)	−0.03 (−0.71–0.65)	0.03 (−0.28–0.35)	0.16 (−0.12–0.43)
Factory worker	0.50 (−1.44–2.45)	0.31 (−1.18–1.80)	0.15 (−1.05–1.34)	0.02 (−1.06–1.10)	−0.05 (−0.61–0.50)	0.10 (−0.57–0.76)	0.09 (−0.22–0.40)	0.08 (−0.20–0.35)
Other	−0.09 (−1.81–1.62)	−0.01 (−1.31–1.30)	−0.09 (−1.14–0.96)	−0.25 (−1.20–0.70)	−0.23 (−0.72–0.25)	−0.03 (−0.61–0.56)	0.08 (−0.19–0.36)	0.00 (−0.24–0.24)

CI= confidence interval.

Table S2. Significant results of logistic regression models for pesticides with detection rates of 10%–50%, analyzing household, parental, and seasonal characteristics as predictors of exposure.

	**Dichotomous Pesticide Results**
**Predictor (Referent)**	**OR (95% CI)** [1]
# People in home	Mirex: 0.83 (0.70–0.99)
Income (≥100,000 Yuan)	
30,000–49,999 Yuan	Omethoate: 0.44 (0.22–0.90)
Maternal age	DEDTP: 0.92 (0.86–0.98)
	Carbophenothion sulfone: 0.91 (0.84–0.99)
	Mirex: 0.92 (0.86–0.99)
	Metalaxyl: 0.92 (0.86–0.99)
Paternal age	o,p′-DDE: 1.08 (1.01–1.15)
Maternal education (College)	
Middle school or less	2,4-Dichlorophenoxyacetic acid: 2.02 (1.09–3.75)
Paternal education (College)	
High school/secondary school	Carbophenothion sulfone: 0.40 (0.16–0.99)
	Oxadixyl: 2.36 (1.22–4.56)
Maternal occupation (Housewife)	
Other	Fluvalinate-tau: 2.07 (1.10–3.90)
	Tetramethrin: 1.78 (1.01–3.14)
Season of birth (Fall/Winter)	
Spring	Omethoate: 2.55 (1.37–4.74)<br>Chlorpyrifos: 0.41 (0.22–0.77)<br>DEDTP: 2.55 (1.37–4.74)<br>Cypermethrin: 0.36 (0.20–0.65)<br>Fenvalerate: 0.32 (0.10–0.98)<br>Tetramethrin: 2.95 (1.42–6.13)<br>o,p′-DDE: 0.43 (0.22–0.85)<br>p,p′-DDE: 2.07 (1.16–3.69)<br>Prothiophos: 0.47 (0.27–0.83)
Summer	Chlorpyrifos: 0.25 (0.13–0.46)<br>Fenvalerate: 0.26 (0.09–0.80)<br>o,p′-DDE: 0.48 (0.24–0.95)<br>Tetrahydrophthalimide: 0.45 (0.21–0.95)
Month of birth (December)	
March	Cypermethrin: 0.12 (0.02–0.71)
April	Cypermethrin: 0.23 (0.07–0.76)<br>Tetramethrin: 6.50 (1.71–24.75)
June	Chlorpyrifos: 0.15 (0.04–0.55)<br>Tetramethrin: 8.25 (1.46–46.60)<br>o,p′-DDE: 0.24 (0.07–0.88)
July	Chlorpyrifos: 0.10 (0.03–0.41)<br>2,4-Dichlorophenoxyacetic acid: 0.16 (0.03–0.82)

[1] Modeled the probability that pesticide <LOD, so a value <1 means higher odds of detection, while a value >1 means lower odds of detection. CI= confidence interval.

© 2015 by the authors; licensee MDPI, Basel, Switzerland. This article is an open access article distributed under the terms and conditions of the Creative Commons by Attribution (CC-BY) license (http://creativecommons.org/licenses/by/4.0/).
